# Clinical Outcomes of Acute Viral Lower Respiratory Infections in Hospitalized Children

**DOI:** 10.7759/cureus.95319

**Published:** 2025-10-24

**Authors:** Margaret A Uchefuna, Giddel Alvarado Castro, Alekya Paripati, Giuliana Colombari Arce, Srushti Patel, Fatima Nasr, Marsha Medows

**Affiliations:** 1 Pediatrics, Woodhull Medical Center, New York City, USA; 2 Pediatrics, New York University School of Medicine, New York City, USA

**Keywords:** bubble cpap and bipap, children under 5 years, clinical severity, high-flow nasal cannula (hfnc), icu admissions, invasive mechanical ventilation, length of hospital stay, lower respiratory tract infection, mechanical ventilation, outcome analysis

## Abstract

Background: Viral infections represent a significant global health strain in children who are below the age of five years. Among these, lower respiratory tract infections (LRTIs) pose a higher risk, particularly in children with comorbid conditions. However, less is known about the impact of LRTIs on healthy children. This academic discourse aims to explore this gap by looking into the clinical outcomes of viral LRTIs in hospitalized children without comorbidities.

Objective: The objective of this study was to investigate whether the clinical outcomes of healthy children below the age of five years hospitalized as a result of viral LRTIs differed depending on the presence of single or multiple viral pathogens.

Methods: We carried out a monocentric retrospective study of 646 children below the age of five years who were admitted for LRTIs at Woodhull Medical and Mental Health Center, New York City, NY, between January 1, 2021, and December 31, 2023. Inclusion and exclusion criteria were applied to select healthy children with no significant medical history. Assessment of statistics was performed using IBM SPSS Statistics software, version 29.0 (IBM Corp., Armonk, NY), to study the clinical outcomes of focus, which included the requirement of a high-flow nasal cannula (HFNC) and other respiratory support, the need for transfer or ICU admission, and the length of hospital stay.* *

Results: The mean age of children in our study was 19.5 months. There were 293 (45.4%) females and 353 (54.6%) males. Of these, 297 (46%) tested positive for a viral pathogen, with respiratory syncytial virus (RSV) being the most common. Post hoc analysis showed a small effect size, Cohen's d = 0.302, 95% CI (0.645, 0.041), and no statistical significance (p = 0.077) when the mean length of hospital stay in children with multiple viruses was compared to those with a single virus. A total of 105 (16.3%) children required pediatric ICU (PICU) transfer, with a higher proportion having multiple viral infections compared to those with a single virus and no virus (p=0.025), with an effect size of 0.176, 95% CI (0.327, 0.076). Of the 68 (10.5%) children requiring respiratory support on admission, the need for support was significantly associated with the presence of a viral infection (p<0.001), although the effect size was also small (0.229) with a 95% CI (0.415, 0.091). There was no significant difference between the type of virus and the need for respiratory support or PICU transfer.

Conclusion: In healthy children below the age of five years hospitalized with viral LRTIs, multiple viral infections were associated with more severe outcomes, including longer duration of hospitalization and a higher likelihood of PICU transfer. These findings call attention to the importance of considering viral co-infections in predicting disease severity and guiding clinical management, even in children with no risk factors.

## Introduction

Lower respiratory tract infections (LRTIs) rank fifth in the leading causes of death worldwide, accounting for about 10% of deaths [1]. LRTIs were the prime cause of death in low-income countries in 2021 [2]. LRTI is an infection that affects the tracheobronchial tree and the alveoli. These infections are comparatively more severe than upper respiratory tract infections, which are usually restricted to above the larynx. In children, common clinical manifestations of LRTIs include bronchiolitis, croup, pneumonia, and exacerbations of wheezing or asthma [3]. The treatment of children with viral LRTIs is usually supportive and may necessitate the use of respiratory assistance like supplemental oxygen, continuous positive airway pressure (CPAP), or mechanical ventilation, depending on the severity of respiratory compromise [4]. 

The burden of LRTIs requiring hospitalization is highest among children below the age of five years, with viruses being the most common cause, sometimes co-existing in multiples, which may complicate management and potentially influence disease severity and clinical outcomes [4]. While some studies have reported that single-virus LRTIs are associated with greater oxygen requirements, longer hospital stays, and increased ICU admissions compared to co-infections, others have found no significant correlation between specific viral pathogens or multiple viral detections and clinical outcomes [5-11].

Clinical outcomes of viral LRTI may be confounded by factors like malnutrition, prematurity, low birth weight, and the presence of existing pulmonary conditions, including asthma and chronic lung disease. We observed that a good number of previous studies did not actively exclude children who may have had some of these conditions that could alter the perceived disease severity, and there is no unanimity on some of the clinical consequences of viral LRTIs in the absence of risk factors. As such, our research intends to answer the question "Do clinical outcomes in children below the age of five years, born at term with normal birth weight and no significant medical history, who were diagnosed with acute viral LRTIs necessitating hospitalization vary with the causative virus?" The clinical outcomes we evaluated included the requirement of high-flow nasal cannula (HFNC) and other respiratory support, the need for transfer or admission to ICU, and the length of hospital stay, all of which reflect disease severity. 

By exploring the relationship between specific viral etiologies and clinical severity, our study aims to bridge an important knowledge gap and provide clinicians with valuable insights that may aid in anticipating patient trajectories and guiding resource allocation in pediatric care settings. 

## Materials and methods

Our study was a monocentric retrospective assessment of 670 electronic medical records from Woodhull Medical and Mental Health Center, New York City, NY. Of the 670 children, 24 children who had missing data were removed; therefore, 646 children were included in the final appraisal. The study focused on hospitalized children below the age of five years diagnosed with acute LTRIs of viral etiology who were admitted between January 1, 2021, and December 31, 2023. We obtained approval from the Office of Science and Research Institutional Review Board of New York University (NYU) Langone Health (study number: i24-00715). Individual authorization requirements were waived, and we took adequate steps to ensure data privacy. No patient names or personal identifiers were used in this study.

Inclusion criteria

Our study included all children below the age of five years who were born at term, with normal birth weight and absence of documented chronic conditions in the medical record, who were diagnosed with acute viral LRTI necessitating hospitalization.

Exclusion criteria

We excluded children with malnutrition, positive blood culture, chronic lung diseases, congenital heart disease, immunodeficiency, low birth weight, or gestational age less than 37 completed weeks.

The objective of our appraisal was to find out if the clinical outcomes in healthy children with viral LRTIs varied with the presence of a single or multiple viruses. The clinical outcomes of focus were the requirement of a HFNC and other respiratory support like bilevel positive airway pressure (BiPAP) and mechanical ventilation; the need for transfer or ICU admission as defined by a worsening clinical condition or requirement of BiPAP or mechanical ventilation; and length of hospital stay. Relevant data were extracted and tabulated for analysis. Assessment of statistics was performed using IBM SPSS Statistics software, version 29.0 (IBM Corp., Armonk, NY). The chi-square and analysis of variance tests were used for qualitative variables, while the two-sample t-test was used for continuous variables.

## Results

**Figure 1:**
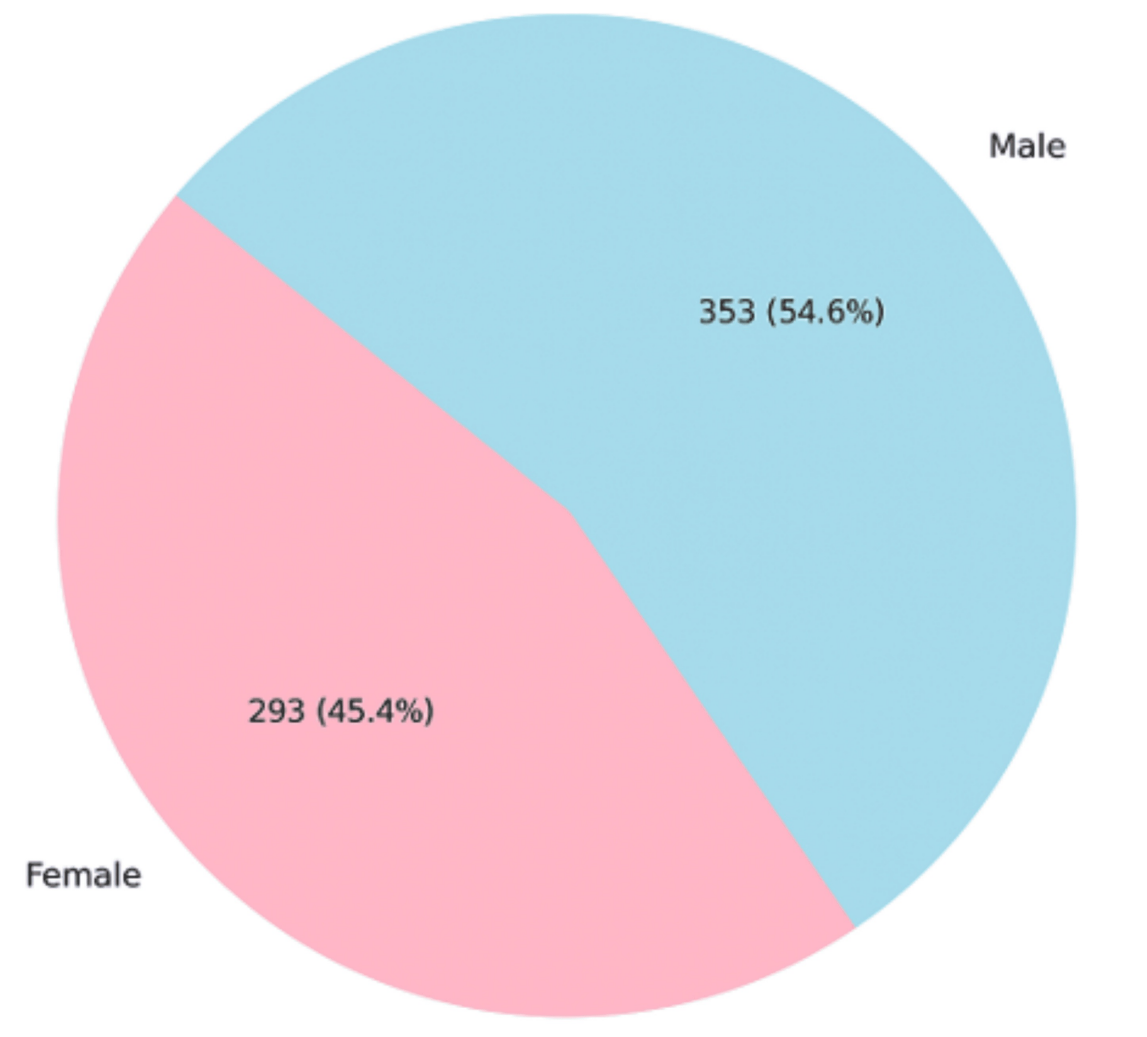
Gender-wise distribution of pediatric patients (n=646)

The mean patient age was 19.5 months (SD= 13.632 months), while the median age was 15.0 months with an interquartile range of 22 months (29 - 7). The majority of the patients (60%) were of Hispanic/Latinx origin. There was a roughly equal gender distribution with 293 (45.4%) female and 353 (54.6%) male patients, as seen in Figure 1

Distribution of viruses

**Figure 2:**
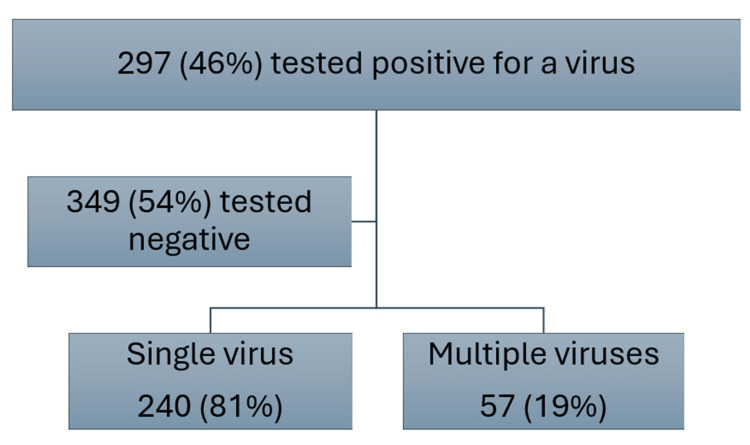
Distribution of cases as per detection of a single virus vs. multiple viruses

Of the 646 children, 297 (46%) had a positive test on the respiratory viral panel, while 349 (54%) had a negative test result. Of the 297 who tested positive for a virus, 240 (81%) had a single virus detected, while 57 (19%) had multiple viruses (two or more) detected, as shown in Figure 2. 

**Figure 3:**
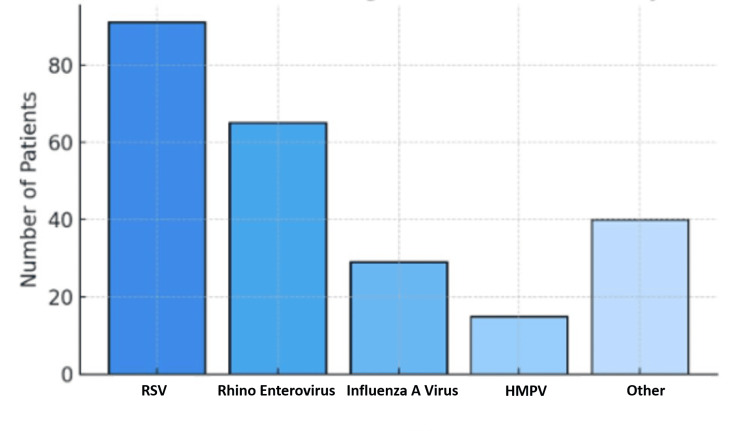
Distribution of patients with single virus infections (n=240) by virus type RSV: respiratory syncytial virus; HMPV: human metapneumovirus

Among the patients with single viruses, the most common virus was respiratory syncytial virus (RSV), which was seen in 91 (38%) patients, followed by rhino enterovirus in 65 (27%) patients, influenza A in 29 (12%) patients, human metapneumovirus in 15 (6%) patients, and other viruses in 40 (17%) patients, as shown in Figure 3.

Length of stay

**Figure 4:**
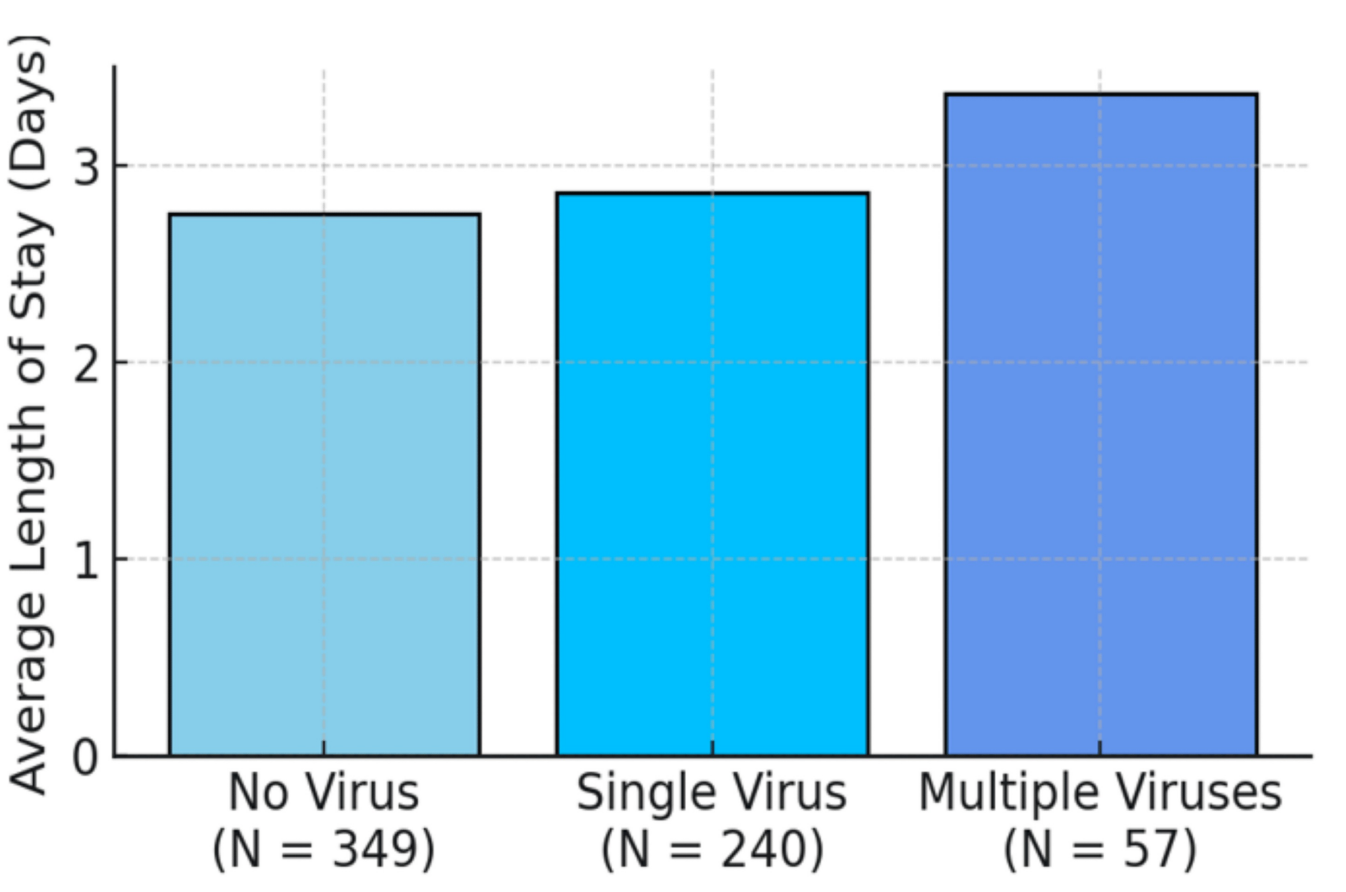
Average length of stay distributed by virus group

The average length of stay in days was 2.75, 2.86, and 3.36 for patients with no virus, a single virus, and multiple viruses, respectively, as shown in Figure 4. 

A post hoc analysis showed a small effect size, Cohen's d = 0.302, 95% CI (0.645, 0.041), and no statistical significance (p = 0.077) when the mean length of hospital stay in children with multiple viruses was compared to those with a single virus, as shown in Table 1.

**Table 1:** Difference in length of stay as a function of the number of viruses

Length of stay in days	Single virus	Multiple virus	t-value	p-value
Mean	2.86	3.36	-1.736	0.077
Standard deviation	1.698	1.376		

Respiratory support on admission

Of the 68 (10.5%) patients who were provided some form of respiratory support at the time of admission due to increased work of breathing or desaturation, 10 (14.7%) required a simple nasal cannula, 50 (73.5%) required a HFNC, and eight (11.8%) required BiPAP, as shown in Table 2.

**Table 2:** Need for respiratory support on admission HFNC: high-flow nasal cannula; BiPAP: bilevel positive airway pressure

Respiratory support on admission	Frequency (n=68)	Percentage (%) of patients who required respiratory support on admission (n=68)
Simple nasal cannula	10	14.7
HFNC	50	73.5
BiPAP	8	11.8

There was also a small effect of 0.22, 95% CI (0.415, 0.091), but a statistically significant relationship between the presence of virus and the need for respiratory support upon admission (χ^2^ = 27.458, p = <0.001) as shown in Table 3.

**Table 3:** Relationship between the number of viruses and the need for respiratory support on admission

Respiratory support on admission	Single virus	Multiple virus	No virus	Chi-square	p-value
Yes	43	16	9	27.458	<0.001
No	197	41	340		

ICU transfer

Of the 646 patients, 105 (16.3%) required transfer to the pediatric ICU (PICU). Of the transfers, 14 (13.3%) had no virus detected, 39 (37.1%) had a single virus, and 52 (49.6%) had multiple viruses detected with an effect size of 0.176, 95% CI (0.327, 0.076). Statistically, there was a significant relationship between the presence of multiple viruses and the need for ICU transfer (χ² = 7.360, p = 0.025), as shown in Table 4.

**Table 4:** Relationship between the number of viruses and the need for ICU transfer

ICU transfer	Single virus	Multiple virus	No virus	Chi-square	p-value
Yes	39	52	14	7.360	0.025
No	201	5	335		

Escalation of care within 24 hours of admission

Of the 578 hospitalized patients who did not require respiratory support on admission, 34 (5.9%) of them needed escalation of care within 24 hours of admission, with nine (26.5%) needing a simple nasal cannula, 14 (41.2%) needing a HFNC, 10 (29.4%) needing BiPAP, and 1 (2.9%) requiring intubation and mechanical ventilation, as shown in Table 5.

**Table 5:** Type of respiratory support required within 24 hours of admission. HFNC: high-flow nasal cannula; BiPAP: bilevel positive airway pressure

Respiratory support within 24 hours of admission	Frequency (n=34)	Percentage (%) of patients who required respiratory support within 24 hours (n=34)
Simple nasal cannula	9	26.5
HFNC	14	41.2
BiPAP	10	29.4
Mechanical ventilation	1	2.9

There was no significant difference between the number of viruses and the need for escalation of care within 24 hours of admission (χ^2^ = 3.693, p=0.158) as shown in Table 6.

**Table 6:** Relationship between the number of viruses and the need for escalation of care within 24 hours of admission.

Escalation of care within 24 hours of admission	Single virus	Multiple virus	No virus	Chi-square	p-value
Yes	13	12	9	3.693	0.158
No	184	29	331		

## Discussion

This retrospective observational work sought to analyze the clinical outcomes and common viral etiologies of LRTIs in children below the age of five years who were admitted to the pediatric inpatient unit of a community hospital in Brooklyn, NY. 

Respiratory viruses, especially RSV and human metapneumovirus, are among the main causes of hospitalization and ICU resource utilization [2,12-15]. Current literature suggests that the implementation of vaccines, improvements in pediatric nutrition, and the COVID-19 pandemic, influenced by prolonged home confinement, decreased peer interaction, and widespread use of face masks, had a declining impact on the presentation, course, and causative pathogens of LRTIs in this age group [2].

The study design specifically excluded children with underlying comorbidities, such as malnutrition, prematurity, chronic lung disease, or immunodeficiencies, in a bid to isolate and better understand the effect of viral LRTIs in otherwise healthy pediatric patients. We also chose a study period that was away from the peak of the COVID-19 pandemic while also excluding children who tested positive for the COVID-19 virus, which, given its novel nature, could cause a unilateral skew to our study results. This methodological approach allowed us to capture a clear picture of how viral respiratory infections affect young children without the confounding influence of preexisting conditions.

With respect to virus frequency, our findings were consistent with the existing body of literature regarding the role of RSV as a major contributor to pediatric LRTIs [2, 12-14]. In our study population, RSV was detected in 38% of admitted patients, reinforcing its status as the dominant viral pathogen associated with a more severe clinical course of LRTI in this age group. However, although human metapneumovirus is believed to be the second most common cause of LRTI hospitalizations in children under five years of age [16], it ranked fourth most common in our study, accounting for only six percent of cases. The second and third most common viruses we saw were rhino enterovirus (27%) and influenza A virus (12%), respectively. 

Our findings revealed that even in the absence of significant comorbidities, approximately one-fifth of study patients still required respiratory support at or within 24 hours of admission and transfer to a higher level of care in the pediatric ICU. Notably, among those who required ICU transfer, the majority tested positive for more than one respiratory virus. This suggests a possible correlation between viral coinfection and increased disease severity, as stated in some prior studies [14,17]. In contrast, patients who tested positive for a single respiratory virus were less likely to require ICU transfer. Similar to other studies, our study did not find any significant difference between specific viral pathogens and the need for escalation of care or PICU transfer [6,18], even though a South German study concluded that RSV mono-infections caused more ICU admissions and longer hospital stays when compared to other viral mono-infections [19].

Another finding in our study was that hospital stay was longer in patients with viral coinfections when compared to single virus infection, although this difference was not statistically significant. This is contrary to a Middle Tennessee study, which concluded that children infected with a single respiratory virus had a greater need for supplemental oxygen, ICU admission, and longer hospitalizations than those infected with multiple viruses [20]. Some other studies posited that there is no difference in length of hospital stay, oxygen requirement, and the need for ICU admissions between single virus infections and coinfections [6, 11, 19].

Some of the limitations of our study were the lack of data on the socioeconomic status of the patients to enable us to know if it had a significant impact on the severity of illness and duration of hospital stay. Also, the viral panel used for our patients was not uniform across the board, as some underwent testing with the Cepheid viral panel (Danaher Corporation, Sunnyvale, CA), which detects about four viruses, while other patients were tested with the GenMark viral panel (Roche Diagnostics, Basel, Switzerland), which detects about 20 viruses. This may explain why some cases identified as having no virus detected still required escalation of care. In addition, it was a single-center study, and due to institutional limitations, patients who required care beyond what our facility could provide, specifically those needing mechanical ventilation or intensive care, were transferred to external tertiary centers, and their eventual clinical outcomes, including mortality, if any occurred, could not be adequately followed up. 

## Conclusions

Our study provides insight into the current trends in pediatric LRTI admissions, highlighting the potential impact of viral coinfections on clinical outcomes and hospital resource utilization even in otherwise healthy children. At the time of this study, our hospital had not yet implemented the RSV prevention protocol for pregnant mothers and their young infants. Therefore, it would be valuable to conduct further studies to gauge the influence of the RSV vaccine on viral LRTI clinical outcomes and to explore whether other viruses might emerge as leading causative agents. Further multicenter studies with long-term follow-up are warranted to more thoroughly appreciate the evolving epidemiology and clinical burden of viral respiratory infections in this young population.
